# Positionspapier der Österreichische Alzheimer Gesellschaft (ÖAG): Auswirkungen der COVID-19-Pandemie in Österreich auf Menschen mit Demenz und deren Betreuungsumfeld – Problemfelder, Empfehlungen und Strategien

**DOI:** 10.1007/s40211-020-00363-9

**Published:** 2020-10-29

**Authors:** Michaela Defrancesco, Christian Bancher, Peter Dal-Bianco, Hartmann Hinterhuber, Reinhold Schmidt, Walter Struhal, Gerhard Ransmayr, Elisabeth Stögmann, Josef Marksteiner

**Affiliations:** 1 Universitätsklinik für Psychiatrie I, Department Psychiatrie, Psychotherapie und Psychosomatik, Medizinische Universität Innsbruck, Anichstraße 35, 6020 Innsbruck, Österreich; 2 Abteilung für Neurologie/neurologische Rehabilitation, Landesklinikum Horn-Allentsteig, Horn, Österreich; 3 Universitätsklinik für Neurologie, Medizinische Universität Wien, Wien, Österreich; 4 Universitätsklinik für Neurologie, Klinische Abteilung für Neurogeriatrie, Medizinische Universität Graz, Graz, Österreich; 5 Abteilung für Neurologie, Universitätsklinikum Tulln, Karl Landsteiner Privatuniversität für Gesundheitswissenschaften, Standort Tulln, Tulln, Österreich; 6 Abteilung für Neurologie, Kepler Universitätsklinikum, Linz, Österreich; 7 Department für Psychiatrie und Psychotherapie A, Landeskrankenhaus Hall, Hall, Österreich

**Keywords:** COVID-19, Pandemie, Demenz, Pflegende Angehörige, Pflegeheim, COVID-19, Pandemics, Dementia, Cargivers, Nursing homes

## Abstract

Ältere Menschen sind durch die COVID-19 Pandemie besonders betroffen. Die meisten der Verstorbenen sind ältere Erwachsene, von denen ein Großteil vorbestehende Gesundheitsprobleme hatten. Weltweit leiden mehr als 50 Mio. – in Österreich etwa 140.000 Menschen an Demenz. Demenz hat sich in einer alternden Gesellschaft zu einer Pandemie entwickelt. Der Doppelschlag von Demenz- und COVID-19-Pandemien hat bei diesen Menschen und deren Angehörigen große Besorgnis ausgelöst. Die Covid-19-Pandemie stellt Patienten mit Demenz und deren Angehörige vor große Herausforderungen: 1. durch begrenzten Zugang zu genauen Informationen über die COVID-19-Pandemie, Schwierigkeiten, sich an Schutzverfahren, wie das Tragen von Masken, zu erinnern oder die ihnen zur Verfügung gestellten Informationen zu verstehen. 2. Demenzpatienten leben allein oder mit ihrem Ehepartner, ihren Bezugspersonen zu Hause oder in Pflegeheimen. Um die Ansteckungsgefahr in Pflegeheimen zu verringern, wurden Besuche in Pflegeheimen und Langzeitpflegeeinrichtungen verboten. Sozialdistanzierende Maßnahmen sind flächendeckend eingesetzt worden. Folglich verloren ältere Bewohner den persönlichen Kontakt zu ihren Familienmitgliedern und wurden sozial isoliert. Auch Gruppenaktivitäten in Pflegeheimen wurden verboten. Es wurde beobachtet, dass unter dem doppelten Stress der Angst vor Infektionen und der Sorge um den Zustand der Bewohner das Angstniveau unter dem Personal in den Pflegeheimen im Verlauf der vollständigen Abschottung zunahm und Anzeichen von Erschöpfung und Burnout auftraten. Die Österreichische Alzheimer Gesellschaft (ÖAG), wie auch bereits andere internationale Gesellschaften, möchten mit diesem Artikel aufgrund der akuten COVID-19-Krise Menschen mit Demenz und ihr Betreuungsumfeld unterstützen. Neben dem physischen Schutz vor Virusinfektionen sollten auch Empfehlungen für die psychische Gesundheit und Möglichkeiten der psychosozialen Unterstützung auf verschiedenen Ebenen aufgezeigt werden.

## Abstract (en)

Older adults are particularly affected by the current COVID-19 (SARS-CoV-2) pandemic. The risk of dying from COVID-19 increases with age and is often associated with pre-existing health conditions. Globally, more than 50 million—in Austria currently approximately 140,000 people—suffer from dementia. The co-occurrence of dementia as a “pandemic of old age” together with the COVID-19 pandemic has a double impact on persons living with dementia and their caregivers. The COVID-19 pandemic poses major challenges for individuals with dementia and their caregivers: (1) People with dementia have limited access to information on COVID-19, may have difficulties with protective measures such as wearing masks and in remembering safety regulations. (2) People with dementia live alone or with their family, or are institutionalized. To reduce the chance of infection among older people in nursing homes, Austrian local authorities have banned visitors to nursing homes and long-term care facilities and implemented strict social-distancing measures. As a result, older people lost face-to-face contact with their family members, became isolated and social activities stopped. Consequently, anxiety, stress and serious concerns about infections among staff in nursing homes increased and they developed signs of exhaustion and burnout during the full lockdown of the facilities. Thus, due to the emerging COVID-19 crisis, the Austrian Alzheimer Association (Österreichische Alzheimer Gesellschaft, ÖAG) and international societies developed recommendations to support people living with dementia and their caregivers on various issues of physical and mental health.

## Einleitung

Am 31.12.2019 wurde erstmals auf der Homepage der WHO folgende Meldung veröffentlicht: *„Am 31. Dezember 2019 wurde das WHO-Länderbüro in China darüber in Kenntnis gesetzt, dass in der Stadt Wuhan in der chinesischen Provinz Hubei mehrere Fälle von Lungenentzündung unbekannter Ursache aufgetreten sind. Die chinesischen Behörden haben bei einer mit Lungenentzündung ins Krankenhaus eingelieferten Person vorläufig ein neuartiges Corona-Virus identifiziert.“* (http://www.euro.who.int/de/health-topics/health-emergencies/coronavirus-covid-19/news/news/2020/01/novel-coronavirus-emerges-in-china). Am 07.01.2020 wurde das neuartige Corona-Virus identifiziert und als COVID-19 bezeichnet. Seither breitete sich COVID-19 weltweit aus und wurde am 30.01.2020 von der WHO als „gesundheitliche Notlage von internationaler Tragweite“ und schließlich am 12.03.2020 zu Pandemie erklärt. 

In Europa wurde erstmals am 24.01.2020 von drei bestätigten COVID-19 Patienten in Frankreich berichtet. Am 02.05.2020 wurden in Europa 1.493.483 Mio. bestätige Fälle und 140.620 COVID-assoziierte Todesfälle gemeldet (https://who.maps.arcgis.com/apps/opsdashboard/index.html#/ead3c6475654481ca51c248d52ab9c61). Die erschreckenden Erfahrungen aus China und auch den Zentren der Pandemie in Europa wie Italien, Frankreich und Spanien zeigten deutlich, dass vorwiegend Menschen ab dem 60. Lebensjahr und insbesondere jene mit Vorerkrankungen wie Diabetes, kardiovaskulären Erkrankungen, Lungenerkrankungen und geschwächtem Immunsystem zur Hauptrisikogruppe für eine Infektion mit tödlichem Ausgang zählen [1]. Menschen mit Demenz – vorwiegend vom Alzheimer-Typ oder gemischt vaskulären Typ – zeigen in den meisten Fällen die genannten Risikofaktoren [2] und fallen somit unter die Hochrisikogruppe im Rahmen der COVID-19-Pandemie. In Anbetracht des besonderen Risikos für Menschen mit Demenz, sollte insbesondere gerade dieser Gruppe von Menschen besondere Aufmerksamkeit geschenkt werden. Auch wenn die COVID-19-Pandemie weltweit mehr als 30 Mio. Menschen betrifft und mehr als 900.000 Menschen an den Folgen des Virus starben (Stand 24.09.2020, WHO) zeigen auch dementielle Erkrankungen Ähnlichkeit zu einer Pandemie mit stark steigender Prävalenz. Im Gegensatz zu COVID-19 liegt die Prävalenz demenzieller Erkrankungen immerhin bei über 50 Mio. weltweit (*World Alzheimer Report 2019* [3])*. *Auch wenn beide Entitäten weder aus ethischer noch aus medizinischer Sicht miteinander verglichen werden können, betreffen beide gemeinsame Risikogruppen und gelten als schlecht bis nicht kausal behandelbar. Fokussierend auf Menschen mit Demenz sind beide – die COVID-19 Pandemie und Demenz – nicht als nebeneinander bestehend, sondern als sich gegenseitig beeinflussende und verstärkende Entitäten anzusehen. In diesem Positionspapier sollen die Folgen und Auswirkungen der COVID-19-Pandemie auf die besonders vulnerable Gruppe der Menschen mit Demenz und deren Betreuungsumfeld auf unterschiedlichen Ebenen dargestellt und kritisch diskutiert werden. Trotz der dramatischen Folgen der Pandemie für Menschen wie auch Wirtschaft sollte diese Krise auch als Chance gesehen werden. Jedenfalls soll sie Anlass sein, bestehende Versorgungs- und medizinische Strukturen für Menschen mit Demenz in Österreich zu beleuchten, zu hinterfragen und auch in Anbetracht noch folgender Krisen neu zu evaluieren.

### Beginn der Pandemie in Österreich

In Österreich wurden am 01.03.2020 lediglich 14 Personen positiv auf COVID-19 getestet – https://www.oesterreich.gv.at/?gclid=EAIaIQobChMI1_HhlKuD6QIVgbHtCh1hVAoQEAAYASAAEgKG2PD_BwE. Bis 04.05.2020 stieg die Zahl der bestätigten COVID-19 Fälle auf 15.535 an. Laut den veröffentlichten epidemiologischen Zahlen des österreichischen Gesundheitsministeriums (https://info.gesundheitsministerium.at) erreichte die Ausbreitung der COVID-19-Infektionen gemessen an den bestätigten Neuerkrankungen am 26.03.2020 und somit 10 Tage nach dem Inkrafttreten des COVID-19-Maßnahmengesetzes seinen ersten Höhepunkt. Dennoch stieg die Zahl der Todesfälle von 71 (26.03.2020) bis auf 762 (24.09.2020) nach einem zwischenzeitlichen Rückgang der Infektionen zwischen Mai und Juni 2020 erneut weiter an. Insbesondere die Todesfälle je Altersgruppe spiegeln das hohe Risiko und die Gefahr für die ältere Bevölkerung und somit auch die Gruppe von Menschen mit hoher Demenzprävalenz wider. In die Altersgruppe >64 Jahren fielen 490 (91 %) der gemeldeten Todesfälle. Ab diesem Alter beginnt auch die Prävalenz von demenziellen Erkrankungen, insbesondere der Demenz vom Alzheimer-Typ, anzusteigen. Während in der Altersgruppe zwischen 64 und 70 Jahren noch von einer Prävalenz von 1,5 % auszugehen ist – steigt diese Prävalenz auf über 44 % in der Altersgruppe über 95 Jahre an. Allein 206 (38 %) der COVID-19-assoziierten Todesfälle fielen in die Altersgruppe >84 Jahre. Nach Daten aus Metaanalysen leiden in dieser Altersgruppe 12,8–22,2 % der Menschen an einer Form von Demenz [4, 5]. Nationale Daten zur Anzahl von Todesfällen von Menschen mit Demenz in Österreich liegen derzeit noch nicht vor. Internationale Daten von Spanien und Italien (http://www.euro.who.int/de/health-topics/health-emergencies/coronavirus-covid-19/weekly-surveillance-report) berichten, dass 24 % der COVID-19-assoziierten Todesfälle unter einer komorbiden neurologischen Erkrankung oder Demenz litten. Seit April 2020 wird basierend auf diese Daten in einer zunehmenden Anzahl von Publikationen Stellung zum Umgang mit Menschen mit Demenz und dem Betreuungsumfeld im Rahmen der COVID-19-Pandemie bezogen. In mehreren Editorials und Kommentaren wird explizit auf die besonderen Bedürfnisse dieser vulnerablen Population hingewiesen [6–10]. In einem Artikel von Brown et al. 2020 [11] wurden bereits im April Empfehlungen veröffentlicht, die sich mit dem Umgang mit Menschen mit Demenz im stationären Setting, der psychischen Belastung für Angehörige und auch die Auswirkungen der COVID-19-Pandemie auf die Demenzforschung beschäftigen.

#### Beginn der Maßnahmen

Nach dem Vorbild von China hat man in Österreich entschieden, die Ausbreitung von COVID-19 durch Reduktion von sozialen Kontakten, Einschränkungen des öffentlichen Lebens und Kundmachung von hygienischen Maßnahmen einzudämmen. Der § 2 Z 1 des COVID-19-Maßnahmengesetzes trat mit 16.03.2020 in Kraft. Für die Bevölkerung waren besonders die folgenden sog. „Ausgangsbeschränkungen“ relevant, da sie maßgeblich die Rechte und Freiheit der Bevölkerung einschränkten.

COVID-19-Maßnahmen: Es gab nur 4 Gründe, das Haus zu verlassen:

1. um zur Arbeit zu gehen, wenn das notwendig ist,
2. für dringend notwendige Besorgungen,
3. um anderen Menschen zu helfen und
4. in besonderen Ausnahmefällen galt: Wer im dringenden Fall ins Freie muss, soll das ausschließlich allein machen oder mit den Personen, die in der gemeinsamen Wohnung leben.

Zeitgleich wurden in Österreich alle Bildungseinrichtungen und Geschäfte mit Ausnahme von Lebensmittelmärkten (für Mensch und Tier), Drogerien und Apotheken geschlossen. Dienstleistungsbetriebe wie Friseure, Fußpflege oder Masseure durften nichtmehr öffnen und alle Gaststätten wurden geschlossen. Religiöse Feste und Feiern wie Taufen, Messen oder Hochzeiten wurden verboten.

Im medizinischen Bereich wurden Rehabilitationseinrichtungen und Kuranstalten geschlossen, zahlreiche elektive Behandlungen und Ambulanztermine abgesagt und ein Besuchsverbot in Krankenhäusern und Wohnheimen verhängt.

Diese Maßnahmen stellten für alle Bevölkerungsschichten und Personengruppen eine erhebliche Einschränkung des Lebens und meist auch eine erhebliche soziale, psychische und auch ökonomische Belastung dar.

Ab dem 14.04.2020 begann die Regierung, die Maßnahmen schrittweise zurückzunehmen und zu lockern. Es wurden jedoch auch zusätzliche Maßnahmen wie das Tragen eines Mund-Nasen-Schutzes, z. B. in Geschäften oder öffentlichen Verkehrsmitteln, eingeführt. Wann und wie Maßnahmen mit hoher Relevanz für Menschen mit Demenz gelockert werden – beispielsweise das Besuchsverbot in Wohnheimen und Krankenhäusern oder das Öffnen von medizinischen Dienstleistern – ist mit Stand vom 03.05.2020 noch unklar. Einen bitteren Beigeschmack hinterlässt auch die Bekanntgabe der Regierung von Maßnahmen zum Schutz von Risikogruppen am 23.04.2020. Diese beinhalten ausschließlich eine Regelung für den Schutz von Personen mit Vorerkrankungen am Arbeitsplatz. Menschen mit Demenz – eine Personengruppe, die nahezu gänzlich nicht mehr im Arbeitsprozess und aufgrund der Altersstruktur zur Hauptrisikogruppe für eine letale COVID-19-Infektion gehört, – werden durch keine gesonderten Maßnahmen geschützt.

Zusammengefasst bedeuten die COVID-19-bedingten Maßnahmen für Menschen mit Demenz auf verschiedenen Ebenen eine durch ihre Erkrankung spezielle und erhebliche zusätzliche Belastung. Ziel der Maßnahmen war die Verhinderung der Verbreitung von COVID-19 und somit der Schutz der Bevölkerung. Im Folgenden soll dargestellt und diskutiert werden, auf welchen Ebenen diese „Schutzmaßnahmen“ bei Patienten mit Demenz und deren Betreuungsumfeld auch schaden oder weitrechende negative Auswirkungen haben können und möglicherweise noch haben werden. Auch soll aufgezeigt werden, welche Faktoren und teils auch strukturellen demenzbezogenen Gegebenheiten die Umsetzbarkeit der COVID-19-bedingten Maßnahmen bei Menschen mit Demenz schwierig bis nicht realisierbar machen. Nicht zuletzt werden auch die im Rahmen der Krise implementierten gesonderten und speziellen Hilfsangebote für Menschen mit Demenz aufgezeigt.

## Methodik

In diesem Positionspapier wurden aktuelle Informationen aus offiziellen Quellen der österreichischen Regierung, der Weltgesundheitsorganisation (WHO) sowie rezent publizierter Literatur im Rahmen der COVID-19-Pandemie verarbeitet.

Problemfelder wurde anhand von Berichten und aktuellen Erfahrungen aus geriatrischen und gerontopsychiatrischen Einrichtungen in Österreich sowie anhand bekannter Literatur definiert.

Jedes Problemfeld wurde in Bezug auf die aktuelle COVID-19-Krise und in Zusammenschau mit bereits publizierter Literatur analysiert und diskutiert.

## Ergebnisse und Problemfelder

Basierend auf publizierten Originalarbeiten, Fallberichten und Stellungnahmen zur COVID-19-Pandemie zu Menschen mit Demenz und deren Betreuungsumfeld werden allgemeine Auswirkung von Krisen und insbesondere der COVID-19-Krise auf diese vulnerable Population beschrieben. In den Unterkapiteln werden spezielle Problemfelder auf den folgenden 4 Ebenen unter Bezugnahme auf die rezente Literatur diskutiert und vonseiten der ÖAG Stellungsnahmen und Handlungsempfehlungen formuliert:

- Ebene: Versorgung, Betreuung und Pflege
- Ebene: medizinische Ebene
- Ebene: soziale Ebene
- Ebene: kognitive, emotionale und Verhaltensebene

**Figure 1:**
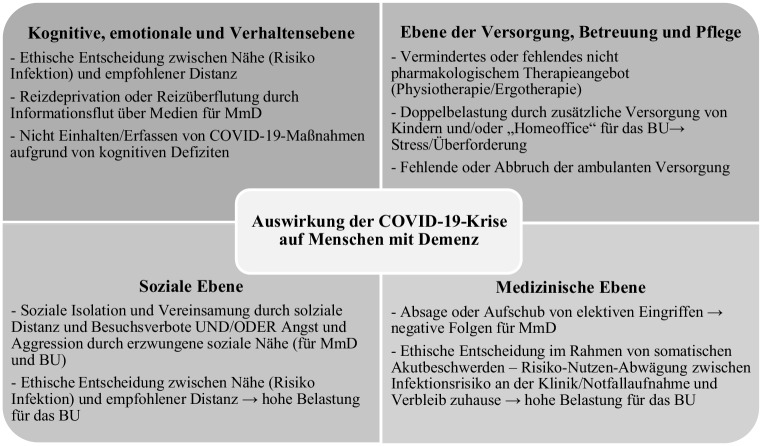

Abb. 1 gibt eine Übersicht über die identifizierten durch COVID-19 neu entstandenen Problemfelder für Menschen mit Demenz und/oder das Betreuungsumfeld auf verschiedenen Ebenen. Für jede Ebene werden wesentliche krisenassoziierte Folgen und Auswirkungen auf Menschen mit Demenz und deren Betreuungsumfeld dargestellt.

### Auswirkung von Krisen auf Menschen mit Demenz

Das Thema „Krise bei Menschen mit Demenz“ ist keineswegs erst mit dem Beginn der COVID-19-Pandemie entstanden. Die Auswirkungen von Krisen auf diese vulnerable Gruppe sind jedoch nur wenig untersucht. In einer Übersichtsarbeit von MacNeil Vrommen et al. – [12] zum Thema „Definition von Krisen in der Pflege von Menschen mit Demenz“ konnten lediglich 27 Publikationen eingeschlossen werden [12]. Die Autoren beschreiben in ihrer Arbeit multiple Auslöser einer Krise im Rahmen der Pflege von Patienten mit Demenz – eine Pandemie vergleichbar mit COVID-19 wurde erwartungsgemäß nicht thematisiert. Dennoch wurde im Artikel eine Definition für „demenzassoziierte Krisen“ formuliert (englisches Original): „*A process where there is a stressor(s) that causes an imbalance requiring an immediate decision which leads to a desired outcome and therefore crisis resolution. If the crisis is not resolved, the cycle continues.“ *Trotz dem neu hinzugekommenen Stressor „COVID-19“ hat diese Definition auch für die rezente Krise nicht an Aktualität verloren. Auch in einer aktuell 2020 publizierten Übersichtsarbeit zum Management von Krisen bei Patienten mit Demenz wurde ein Mangel an entsprechenden Studien und die Notwendigkeit der Entwicklung klarer Krisenkonzepte schlussgefolgert [13]. Als vorrangiges Ziel in den genannten Studien wurde angeführt, Akutaufnahmen und den Transfer an Notfallaufnahmen von Menschen mit Demenz zu vermeiden und ein effektives Krisenmanagement für die Patienten in gewohnter Umgebung zu ermöglichen [14]. Auch die 2018 veröffentlichen NICE Guidlines heben die Wichtigkeit der Pflege von Menschen mit Demenz im gewohnten Umfeld zuhause hervor [15]. Diese Ziele haben im Rahmen der COVID-19-Pandemie einen noch höheren Stellenwert und an maßgeblicher Relevanz gewonnen. Es ist bekannt, dass ein Akuttransfer von Menschen mit Demenz bei den Patienten mit hohem Stress, Angst und Schwierigkeiten, sich in der neuen Umgebung zurechtzufinden, verbunden ist. Im Rahmen der derzeitigen Pandemie kommen noch zwei wichtige Faktoren hinzu, die einen Transfer ins Krankenhaus zu einer äußerst schlechten Strategie des Krisenmanagements machen:

- Menschen sind an Kliniken, Krankenhäusern und Notfallaufnahmen einem erheblich erhöhten Infektionsrisiko mit COVID-19 ausgesetzt.
- Durch das maßnahmenbedingte Besuchsverbot in medizinischen Einrichtung kommt es zu einer zusätzlich belastenden sozialen Isolation der Menschen mit Demenz. Aus diesem Grund sollten insbesondere präventive Maßnahmen im Zentrum eines erfolgreichen Krisenmanagements stehen.

Einige rezente Publikationen enthalten bereits Stellungnahmen und Kommentare zu präventiven Strategien für Menschen mit Demenz und deren Betreuungssystem, um allgemeinen negativen Folgen der Pandemie aktiv vorzubeugen [16–18]. In einer Übersichtsarbeit von Boots et al. 2014 [19] wurden Arbeiten über internetbasierte Interventionsprogramme für Angehörige und Betreuungspersonen von Menschen mit Demenz zusammengefasst. Trotz der wenigen publizierten und der teils methodisch geringen Aussagekraft der Studien wurde ein positiver Effekt auf Pflege und auch die psychische Gesundheit der Pflegenden beschrieben. Gerade in Krisen mit der Notwendigkeit von sozialer Distanz zeigt sich die Relevanz solcher webbasierter, Medien nutzender Interventionsstrategien.

Im Bereich von pharmakologischen Studien steigt seit Beginn der COVID-19-Pandemie vorrangig die Anzahl von wissenschaftlichen Publikationen mit Daten zu möglichen Impfstoffen oder neuen Therapien gegen den COVID-19-Virus. Trotz der dringenden Notwendigkeit und akuten Relevanz solcher Studien, sollten auch die Effekte auf die „Demenz Pandemie“ nicht gänzlich vernachlässigt werden.

Es ist zu erwarten, dass die derzeitige COVID-19-Pandemie auch für die Forschung auf dem Gebiet der Alzheimer-Erkrankung weitreichende Folgen haben wird. Insbesondere die Durchführung von klinischen Studien und Pharmastudien können durch Quarantäne – und umfangreiche Sicherheitsmaßnahmen sowie Reiseverbote, jedoch auch durch die weitreichenden wirtschaftlichen Folgen behindert und erschwert werden. Diesbezüglich wurden bereits einige Richtlinien für die Durchführung von klinischen Studien bei Patienten mit Alzheimer-Erkrankung erarbeitet und publiziert [20]. Eine Fortführung von pharmakologischen Studien unter Einhaltung von hohen Sicherheitsmaßnahmen sollte jedenfalls erfolgen.

### Ebene: Versorgung, Betreuung und Pflege

Menschen mit Demenz werden in Österreich zu ca. 80 % zu Hause und etwa in 15–20 % in einem Wohnheim oder anderen vollstationären Einrichtung versorgt [21]. Die Versorgung zu Hause wird zu einem überwiegenden Teil von Angehörigen allein übernommen – teils auch im Rahmen einer 24-h-Betreuung oder auch mit Unterstützung ambulanter Pflegeeinrichtungen gemeistert. Einen wichtigen Teil der Betreuung und Pflege von Menschen mit Demenz stellen auch nicht pharmakologische Therapiemaßnahmen wie die Ergotherapie, Musiktherapie oder körperliche und kognitive Aktivierung beispielsweise in Tageszentren dar.

Im Rahmen der COVID-19-Maßnahmen wurden zahlreiche ambulante Betreuungsangebote reduziert oder auch Angebote in Tageszentren gänzlich eingestellt. Die Versorgung mithilfe der *24-h-Pflege* wurde über die im Rahmen der Maßnahmen eingeführten Reisebestimmungen und Grenzkontrollen erschwert und musste teils beendet werden. Für Familien mit einem Angehörigen mit Demenz kann eine 24-h-Betreuung eine wertvolle Unterstützung sein. Die Covid-19-Krise führte drastisch vor Augen, wie fragil dieses System ist.

Durch die COVID-19-Krise wurde akut und deutlich der Mangel an einheimischen Pflegekräften aufgezeigt. Auch wenn durch Bemühungen der Regierung Ende April 2020 eine Ein- und Ausreise von Pflegekräften aus osteuropäischen Ländern wieder eingeschränkt ermöglicht wurde, konnte der COVID-19-bedingte akute Pflegenotstand nicht behoben werden.

#### Stellungnahme der ÖAG zur 24-h-Pflege

Es besteht auch nach der COVID-19-Krise ein dringender Bedarf, besonders das Angebot an einheimischen Pflegekräften für Menschen mit Demenz entscheidend zu erhöhen. Das Risiko einer Covid19-Infektion für Pflegekräfte, die über lange Strecken anreisen, ist erhöht. Auch lässt sich das Infektionsrisiko in ihren Herkunftsregionen meistens nicht erfassen. Entsprechend muss die Autonomie der österreichischen 24-h-Pflege über bessere Entlohnung und größere Wertschätzung verbessert werden.

Nach Auskunft von *ambulanten Pflegeeinrichtungen* war die Reduzierung im Bereich der ambulanten Pflege und Versorgung von Menschen mit Demenz im Wesentlichen durch die hohe Gefahr der Infektion der zu betreuenden Klienten über die Pflegekräfte begründet. Diese mussten sich einer ethisch schwierigen und belastenden Risiko-Nutzen-Abwägen zwischen Fortführung der Betreuung und Risiko einer Infektion des Klienten stellen. Der Umstand, dass insbesondere Körperpflege ohne körperliche Nähe nicht durchführbar ist, machte eine solche oft unmöglich. Hier ist besonders auf das Fehlen von Schutzkleidung für ambulante Pflegepersonen hinzuweisen. Mit einer ausreichenden Schutzbekleidung und FFP 3 Masken wäre in einem größeren Ausmaß eine Weiterführung der ambulanten Betreuungs- und Pflegemaßnahmen ermöglicht worden.

#### Stellungnahme der ÖAG zu ambulanter Pflege

Es wird der dringende Aufruf an die Regierung gestellt, auch ambulante Pflegeeinrichtungen vorsorglich mit medizinischem Schutzmaterial zu versorgen. Auch sollte die COVID-19-Pandemie zum Anlass genommen werden, Hygieneartikel wie Hände- und Flächendesinfektionsmittel auch für die ambulanten Dienste zur Verfügung zu stellen, die Pflegepersonen in deren Anwendung zu schulen und die Desinfektion von Händen und Flächen in die Pflegetätigkeit zu integrieren.

Für *pflegende Angehörige* stellten insbesondere die Ausgangsbeschränkungen und die Vorschrift zum Einhalten von sozialer und räumlicher Distanz eine große Herausforderung und Belastung dar. Pflegende Angehörige, die nicht im selben Haushalt mit dem Menschen mit Demenz leben, waren gezwungen, auf den gewohnten Kontakt durch regelmäßige Besuche oder Abholen dieser zu Ausflügen etc. zu verzichten. Die pflegenden und betreuenden Angehörigen wurden zu einer schwierigen auch ethischen Risiko-Nutzen-Abwägung zwischen bestmöglicher Betreuung und Pflege des Betroffenen und bestmöglichem Schutz vor Infektion gezwungen.

Eine alternative Kontaktaufnahme mittels Videotelefonie oder anderen sozialen Medien ist bestenfalls bei Menschen mit leichtgradiger Demenz in beschränktem Ausmaß möglich, in schwereren Stadien oder somatischen und/oder psychiatrischen Komorbiditäten unrealistisch.

Pflegende Angehörigen sind auch unabhängig von der COVID-19-Krise neben den Menschen mit Demenz selbst eine vulnerable Gruppe mit hohem Risiko für physischen und emotionalen Stress und assoziierten Folgeerkrankungen [22, 23]. Die telemedizinische oder internetbasierte Unterstützung kann für Angehörige ein wichtiges und hilfreiches Angebot darstellen. Der Nutzen von Internet und telemedizinischen Unterstützungsangeboten für pflegende Angehörige wurde bereits vor der COVID-19-Krise untersucht [19]. Frühere Studien führten als entscheidenden Vorteil der telemedizinischen und internetbasierten Betreuung und Beratung von Angehörigen an, dass diese von zuhause aus erfolgen kann und der zu betreuende Angehörige weniger Isolation empfindet. In Zeiten von Ausgangsbeschränkungen und „sozialem Distanzhalten“ wurden diese genannten Vorteile zu einer akuten Notwendigkeit. Auch unabhängig von der Rolle als pflegender Angehöriger stellt die COVID-19-Epidemie für auch gesunde Menschen ein hohes Ausmaß an Stress dar. Auch in der Allgemeinbevölkerung ist wie bereits nach der SARS-Epidemie 2003 (schweres akutes respiratorisches Syndrom) mit dem Auftreten von psychischen Symptomen wie Angst, depressiven Symptomen bis zu selbstschädigendem Verhalten oder Suizidideen zu rechnen [24].

#### Stellungnahme der ÖAG zu pflegenden Angehörigen

Pflegende Angehörige benötigen vergleichbar mit professionellen Pflegenden und Betreuungspersonen einen Zugang zu Schutzmaßnahmen (FFP-3-Masken, Desinfektionsmitteln), um die familiäre Versorgung von Menschen mit Demenz auch in Krisenzeiten sicher gewährleisten zu können. Aus dieser Krise soll gelernt werden, wie wichtig Schulungs- und Unterstützungsangebote für pflegende und betreuende Angehörige auch im Bereich der medizinischen Versorgung sind. Als Konsequenz muss das Angebot solcher Schulungsangebote und auch insbesondere telemedizinischer und internetbasierter Unterstützungsangebote für pflegende Angehörige ausgeweitet werden. Es muss auf die psychische Gesundheit von pflegenden Angehörigen proaktiv geachtet werden, um psychischen Störungen durch die Doppelbelastung durch die COVID-19-Krise auf die Personen selbst und in ihrer Rolle als Pflege- und Betreuungspersonen von Menschen mit Demenz vorzubeugen.

In Anlehnung an die Empfehlungen des Chinesischen Ministeriums für zivile Angelegenheiten im Januar 2020 [25] und jenen vom „US Centers for Disease Control and Prevention“ wurde auch in Österreich ab dem 16.03.2020 ein *Besuchsverbot in Wohnheimen und Betreuungseinrichtungen* erlassen. Dieses Verbot stellt für Menschen mit Demenz auf verschiedensten Ebenen eine durch die demenzielle Erkrankung verstärkt hohe Herausforderung und Belastung dar. Menschen mit Demenz in institutioneller Pflege befinden sich zumeist in einem mittel- bis schwergradigen Krankheitsstadium. – Meist liegen auch andere somatische Komorbiditäten vor. Für diese Patientengruppe stellt ein akuter Wechsel der Tagesstruktur und gewohnter Abläufe ein hohes Ausmaß an Stress und Irritation dar. Die Betroffenen sind aufgrund ihrer kognitiven Defizite nichtmehr in der Lage, sich an neue Gegebenheiten zu adaptieren. Auch können sie Informationen wie die teils komplexe Berichterstattung in den Medien nichtmehr auffassen, verarbeiten und konsolidieren. Insbesondere die demenztypischen Defizite im Neugedächtnis und der Informationsverarbeitung machen eine Anpassung an die neuen Umstände rund um die COVID-19-Krise schwierig bis unmöglich. Die Umstellung von persönlichen Kontakten auf „digitale“ Optionen ist nicht nur durch das häufige Fehlen technischer Geräte, sondern besonders durch die demenzassoziierten Defizite nicht möglich.

Aus den Rückmeldungen der gerontopsychiatrischen Abteilungen und Spezialambulanzen (Gedächtnissprechstunden, Gedächtnisambulanzen) der Bundesländer wurden folgende Problemfelder z. B. aus telemedizinischen Betreuungsangeboten erkannt. Unabhängig wurden auf zahlreichen nationalen und internationalen Internetplattformen zum Thema Demenz eigene Informationen zum Thema „Corona und Demenz“ eingefügt. (Tab. 1).

**Table 1:** 

COVID-19-assoziierte Problemfelder und Berichte aus den Bundesländern	Angebotene Gegenmaßnahmen: – beispielsweise:
Fehlende Betreuung und Pflege, – insbesondere im Bereich der 24-h-Pflege	Einrichtung von offiziellen Hotlines
Unsichere Medikamenteneinnahme durch fehlende ambulante Betreuung	*Alzheimer’s Disease International*: https://www.alz.co.uk/news/adi-offers-advice-and-support-during-covid-19
Mangelhafte Grundversorgung, z. B. hinsichtlich Ernährung aufgrund von geschlossenen Gastbetrieben und Kantinen	MAS Alzheimerhilfe: https://www.alzheimer-hilfe.at/mas_tipps.html#infoblaetter Ehrenamtliche Versorgung für Menschen mit Demenz wurde etabliert – Übernahme von Einkäufen und Botengängen
Fehlendes ambulantes Therapieangebot über Dienstleister wie Physiotherapeuten, Ergotherapeuten	–
Fehlen von Tagesstruktur und körperlicher/geistiger Aktivierung durch die Schließung von Tageszentren und Gruppenveranstaltungen	Coronavirus: Online-Pflege-Kurs & Tipps für die Psyche (öffentliches Gesundheitsportal Österreich: https://www.gesundheit.gv.at/aktuelles/coronavirus-pflege-psyche)
Schwierige Risiko-Nutzen-Abwägung zwischen engmaschigen Besuchen beim zu pflegenden Patienten und Distanzhalten zum Schutz vor Infektion	Tägliche und umfassende Information über nationale und internationale Medien. Einrichtung von offiziellen Hotlines
Anstieg von Angst, Aggression und Agitation in Wohnheimen (auch wegen hoher Covid-19-bedingter Sterberate der Heimbewohner/Heimbewohnerinnen)	Angebot von zahlreichen telemedizinischen psychologischen Beratungsangeboten

### Medizinische Ebene

Auf der medizinischen Ebene und auch im Rahmen der medizinischen Versorgung haben sich für Menschen mit Demenz durch COVID-19 verschiedenste Belastungen und Schwierigkeiten ergeben.

Neben zahlreichen abgesagten und verschobenen elektiven Behandlungen wurden auch ambulante Behandlungsmöglichkeiten vielfach reduziert oder gänzlich auf eine telefonische Erreichbarkeit umgestellt. Trotz des wichtigen und wertvollen Beitrags der telemedizinischen Versorgung während der COVID-19-Krise muss wiederum der eingeschränkte oder fehlende Nutzen für Menschen mit Demenz hervorgehoben werden. Nicht wenige pflegerisch und ärztliche Mitarbeiter werden sich in dieser Krise die immer wieder diskutierten technischen und computerassistierten Möglichkeiten für die Versorgung von Menschen mit Demenz – Schlagwort „Pflegeroboter“ – herbeigewünscht haben. Dennoch zeigt sich in der derzeitigen Situation gerade der hohe Stellenwert von sozialen Kontakten für Menschen mit Demenz.

In der stationären Versorgung ist für Menschen mit Demenz besonders schwer zu verstehen, warum sie sich ohne Menschen, die sie lieben, an einem ihnen unbekannten Ort aufhalten. Sie werden sogar noch einsamer und verängstigter sein als andere. Auch sind sie weniger in der Lage, zu kommunizieren oder Anweisungen und Sicherheitsmaßnahmen zu befolgen. All diese Faktoren können dazu führen, dass sie während ihres Krankenhausaufenthalts ein erhöhtes Risiko haben, ein Delir zu entwickeln. Wie im Abschnitt über ein erfolgreiches Krisenmanagement für Menschen mit Demenz beschrieben, sollte insbesondere in der Zeit von COVID-19 eine strenge Nutzen-Risiko-Abwägung erfolgen, inwieweit ein Krankenhausaufenthalt unvermeidbar ist oder alternative Versorgungsmöglichkeiten genutzt werden können.

In welchem Maße das Covid-19-Virus Einfluss auf mögliche neurologische Symptome und auf die Inzidenz und Prävalenz von Demenzerkrankungen hat, ist derzeit nicht sicher zu beurteilen.

Mit der wachsenden Zahl an Covid-19-Patienten werden bei diesen aber immer mehr neurologische Symptome bekannt. COVID-19 kann das Nervensystem über vier potenzielle Mechanismen beeinflussen, die sich überschneiden können. Der erste Mechanismus ist eine direkte virale Schädigung des Nervengewebes, wie sie bei der Herpes-simplex-Enzephalitis auftritt. Obwohl es einige suggestive Fallberichte gibt, gibt es keinen eindeutigen Beweis dafür, dass das SARS-CoV-2-Virus das zentrale Nervensystem (ZNS) direkt schädigt [26]. Die zweite Art von Verletzung resultiert aus einer exzessiven Immunantwort in Form eines „Zytokinsturms“. Zytokine können die Blut-Hirn-Schranke überwinden und sind mit einer akuten nekrotisierenden Enzephalopathie verbunden. Es wurde nur ein Fall gleichzeitig mit COVID-19 gemeldet. Der dritte Mechanismus der Schädigung des Nervengewebes resultiert aus unbeabsichtigten Wirkungen der Immunantwort des Wirts nach einer akuten Infektion. Ein Beispiel für diese Art der indirekten neuronalen Schädigung ist das Guillain-Barré-Syndrom (GBS). Es wurde über mehrere Fälle von GBS in Verbindung mit COVID-19 berichtet, aber die Beweise für Ursache und Wirkung sind schwach [27]. Der vierte Mechanismus der indirekten viralen Schädigung resultiert aus den Auswirkungen einer systemischen Erkrankung. Längere Behandlungen auf der Intensivstation können neuropsychiatrische Symptome verursachen. Die meisten Fälle von COVID-19-bezogenen neurologischen Komplikationen scheinen in diese Kategorie zu fallen. In einer retrospektiven Fallserie wurde über eine hohe Inzidenz neurologischer Symptome bei 214 hospitalisierten Patienten mit bestätigter COVID-19-Infektion in Wuhan, China, berichtet [28]. Achtundsiebzig (36,4 %) Patienten hatten Symptome des ZNS (24,8 %), des PNS (8,9 %) oder der Skelettmuskulatur (10,7 %). Die beiden häufigsten ZNS-Symptome waren Schwindel (16,8 %) und Kopfschmerzen (13,1 %), wobei auch über eine akute zerebrovaskuläre Erkrankung, Ataxie, Epilepsie und Bewusstseinsstörungen berichtet wurde. Rezente Fallberichte aus New York, aber auch Berichte aus Österreich, zeigten Fälle von COVID-19-assoziierten zerebrovaskulären Ereignissen [29]. Die längerfristigen Auswirkungen auf Inzidenz und Progredienz von Demenzerkrankungen kann derzeit noch nicht wissenschaftlich beantwortet werden. Von negativen Auswirkungen auf die Demenzprogression oder eine Zunahme der Demenzinzidenz muss bei den berichteten direkten negativen Effekten auf das Gehirn ausgegangen werden.

#### Stellungnahme der ÖAG

Zusammenfassend können wir wohl aus der COVID-19-Krise einerseits schlussfolgern, dass der Ausbau von digitalen und telemedizinischen Medien im Gesundheitssystem hilfreich und wesentlich erweitert werden sollte – andererseits müssen auch die Grenzen dieser „a-sozialen“ Versorgung für Menschen mit Demenz im Fokus bleiben. Es besteht eine dringende Notwendigkeit, auch im Längsschnitt die somatischen und demenzspezifischen Auswirkungen von COVID-19 auf Menschen mit Demenz zu erheben.

### Soziale Ebene

Die COVID-19-Pandemie stellt durch die notwendig gewordenen Schutzmaßnahmen auf der sozialen Ebene für Menschen mit Demenz eine hohe Belastung und Herausforderung dar. Durch das Einhalten von körperlicher Distanz und das sog. „social distancing“ wird das Risiko für Vereinsamung und Reizdeprivation erhöht. Insbesondere die offizielle Empfehlung, Kleinkinder nicht in die Nähe von älteren Menschen zu bringen, schottete Großeltern meist von ihren Enkelkindern ab. Auch werden Menschen mit Demenz von der so wichtigen Ressource für Resilienz – nämlich den sozialen Kontakten und der zwischenmenschlichen Interaktion – abgeschnitten. In vielen bisherigen Studien zu Resilienz bei Menschen mit Demenz wurde gerade der Faktor von sozialer Unterstützung und sozialer Interaktion als wesentlich und wichtig beschrieben [30]. Im Rahmen der Demenz nehmen auch Möglichkeiten zur Nutzung und Entwicklung von Coping-Strategien ab. Während im Jugend- und Erwachsenenalter die Nutzung von sozialen Medien und die digitale Kommunikation als Alternativen für persönliche Sozialkontakte genutzt werden können, stehen diese Wege Menschen mit Demenz, insbesondere im fortgeschrittenen Stadium, meist nicht mehr zur Verfügung. Die COVID-19-Krise kann somit bei Menschen mit Demenz zu großer Einsamkeit führen. Einsamkeit wird definiert: „*als eine unangenehme Erfahrung, welche erlebt wird, wenn ein Individuum einen qualitativen und qualitativen Verlust von sozialen Beziehungen über einen längeren Zeitraum erfährt.*“ Einsamkeit kann in eine soziale und emotionale Ebene unterteilt werden. Die COVID-19-Pandemie und ihre Konsequenzen für das tägliche Leben haben das Risiko für Einsamkeit auf beiden Ebenen entscheidend erhöht. Auf emotionaler Ebene musste der Kontakt mit Menschen mit Demenz sehr stark auf eine verbale Kommunikation z. B. per Telefon reduziert werden. Gerade die nonverbale Kommunikation mit Mimik und Gestik oder auch Berührung hat bei Menschen mit Demenz im Krankheitsverlauf eine zunehmende Bedeutung. Durch Verlust von kognitiven Funktionen können ausschließlich verbal präsentierte Informationen nichtmehr ausreichend verarbeitet und aufgenommen werden – jene mit nonverbalem Inhalt jedoch bis ins schwerstgradige Demenzstadium. Auch das Tragen eines Mund-Nasen-Schutzes erschwert Menschen mit kognitiven Einschränkungen und teils auch Visusminderung die Kommunikation mit ihren Mitmenschen. In Gesprächen mit Angehörigen wurde vielfach berichtet, dass Menschen mit Demenz auch auf Angehörigen mit Mund-Nasen-Schutz ängstlich bis ablehnend reagierten. Einsamkeit auf sozialer Ebene wird durch die COVID-19-Krise einerseits durch die Reduktion vom ambulanten Pflegeangebot, dem Untersagen von Treffen in Gruppen und von Veranstaltungen und auch dem Besuchsverbot in Krankenanstalten und Wohnheimen gefördert. Insgesamt wird in der älteren Bevölkerung je nach Wohnort von einer Prävalenz von Einsamkeit zwischen 20 und 40 % ausgegangen [31]. In einer rezenten Stellungnahme von Armitage et al. [32] wird besonders auf die Gefahr von Einsamkeit und ihren Folgen für die ältere Bevölkerung im Rahmen der COVID-19-Pandemie hingewiesen [32]. Bereits frühere Arbeiten haben gezeigt, dass sich durch Einsamkeit das Risiko für somatische Erkrankungen [33] und psychische Symptome wie Angst und Depression erhöht [34]. Wie auch in der angeführten Stellungnahme sollte die COVID-19-Pandemie zum Anlass genommen werden, aktiv der sozialen Isolation und Einsamkeit älterer Menschen vorzubeugen. Für gesunde ältere Menschen oder Menschen mit Demenz im Beginnstadium können auch telemedizinische und digitale Medien präventiv eingesetzt werden. Für mittel-bis schwergradig an Demenz Erkrankte sollte auf eine Ausweitung von persönlichen und mit sozialer Interaktion verbundenen Strategien fokussiert werden. Auch sollte immer darauf Wert gelegt werden, die Angehörigen und das Betreuerumfeld in die präventiven Strategien mit einzubeziehen.

Während die maßnahmenbedingte soziale Distanz besonders für allein lebende Menschen mit Demenz belastend und problematisch ist, zeigt sich für Menschen, die mit ihrem Partner oder in der Familie leben ein teils konträres Bild. Durch die Quarantänemaßnahmen, die fehlende Möglichkeit des Rückzugs in z. B. Lokale, in Tageszentren, in den Freundeskreis oder auch in die Natur kann es zu einem gezwungenen Maß an sozialer Nähe kommen. Im Rahmen der COVID-19-Pandemie wurde von den Medien und auch psychologischen Stellungsnahmen vorwiegend auf resultierende Konflikte von Eltern und Kindern oder in der Partnerschaft fokussiert, die durch die fehlenden Rückzugsmöglichkeiten und die erzwungene vermehrte gemeinsame Zeit auf engem Raum resultieren. Diese nun intensive und teils ganztägige Konfrontation mit einem im selben Haushalt lebenden an Demenz erkrankten Angehörigen kann ebenfalls zu einer erheblichen Belastung für den Patienten das Betreuungsumfeld führen. Gerade hier besteht dringender Bedarf, aktiv pflegende Angehörige in schwierigen Situationen wie Quarantäne und Ausgangsbeschränkungen aktiv zu unterstützen und zu beraten. Zwar wurden zahlreiche COVID-10-Krisen-Hotlines und telefonische Beratungen eingerichtet, – ob diese jedoch auch im Längsschnitt ausreichend und effektiv waren, werden erst die nächsten Monate zeigen.

#### Stellungnahme der ÖAG

Die COVID-19-Maßnahmen können maßgeblich und erheblich auf der sozialen Ebene zu Belastungen für Menschen mit Demenz und deren pflegende Angehörige führen. Es wurden zahlreiche Gegenstrategien im Rahmen von telefonischen Beratungs-Hotlines und einem breiten internetbasierten Informationsangebot gesetzt. Die geschaffenen Angebote sind vorwiegend für Menschen mit Demenz im leichtgradigen Stadium geeignet und nutzbar. Die Entwicklung von effektiven Interventionen für Menschen im mittel- bis schwergradigen Demenzstadium muss aktiv vorangetrieben werden.

### Kognitive, emotionale und Verhaltensebene

Zu den typischen Symptomen der Alzheimer-Demenz und anderen neurodegenerativen Demenzformen gehören Defizite in der Informationsverarbeitung, der Auffassung und der Erfassung von verbalen Informationen. Auch die Informationsgeschwindigkeit ist im Rahmen der Demenz in Abhängigkeit des Schweregrads reduziert (American Psychiatric Association. Diagnostic and Statistical Manual of Mental Disorders 5th edn). Bis zu 90 % der Menschen mit Demenz leiden im Verlauf der Erkrankung an Verhaltensauffälligkeiten oder neuropsychiatrischen Symptomen [35]. Ein erheblicher Anteil ist auch von komorbiden psychischen Störungen wie Depression, Angst oder Schlafstörungen betroffen [36]. Auch wenn die Folgen der COVID-19-Pandemie auf Menschen mit Demenz und deren Angehörige und Betreuungspersonen noch nicht bestimmt vorausgesagt werden können, ist doch aus den Erfahrungen der SARS-Epidemie 2013 von einem hohen Risiko für negative Konsequenzen für die psychische Gesundheit auszugehen. In Folge der SARS-Epidemie, welche insbesondere Hongkong betraf, wurde von einem Anstieg der Suizidraten von 30 % in der Gruppe der über 65-Jährigen berichtet. Ähnlich wie bei COVID-19 waren auch von SARS überwiegend Personen über 60 Jahre von einem letalen Ausgang der Erkrankung betroffen. Trotz den in Hongkong ohnehin höheren Suizidraten als in westlichen Ländern, muss auch bei uns mit einem Anstieg der Suizide oder suizidalen Krisen in der älteren Bevölkerung gerechnet werden. Auch kam es bei 30–50 % der Menschen, die eine SARS Infektion überstanden hatten, zu persistierenden Angstsymptomen und bei der Gruppe von Mitarbeiten im Gesundheitsbereich zu anhaltendem emotionalen Stress [24]. In einer Studie nach der SARS-Epidemie wurden folgende Faktoren als besonders belastend in der Krise beschrieben: Gefühl der Isolation, Hoffnungslosigkeit, Überflutung mit negativen Nachrichten, Verlust sozialer Integration, unspezifische Angst und das Gefühl, Angehörige zu belasten [37].

Bei Menschen mit Demenz ist der Umgang mit negativen Gefühlen durch die bestehenden kognitiven Defizite zusätzlich erschwert. Insbesondere in Krisen sind hilfreiche Coping-Strategien und die Fähigkeit zur Resilienz bei Menschen mit Demenz oft nur eingeschränkt umsetzbar. Unter dem Begriff Resilienz wird nach Windle *ein dynamischer Prozess der aktiven Auseinandersetzung, Adaptierung und der Bewältigung von stressvollen und traumatischen Erfahrungen* verstanden [38]. Unter Coping wird die Anwendung von Bewältigungsstrategien verstanden, um eine schwierige Lebenssituation zu überstehen. Für Menschen mit Demenz sind insbesondere Sozialkontakte ein wesentlicher die Resilienz stärkender Faktor [39]. Nicht zuletzt hat die kognitive Fähigkeit des sog. „decision making“ im Rahmen der COVID-10-Krise einen wichtigen und hohen Stellenwert für die Gesundheit und das Leben von Menschen mit Demenz bekommen. Die Fähigkeit reflektiert und für sich selbst oder andere Entscheidungen zu treffen, erfordern kognitive Funktionen wie Gedächtnis, Aufmerksamkeit und unterschiedliche frontal-exekutive Funktionen [40]. Vor allem diese kognitiven Funktionen sind im Rahmen der Demenz defizitär. Studien mit an Demenz erkrankten Menschen konnten zeigen, dass deren Fähigkeit zur Entscheidungsfindung reduziert ist [41]. Folglich besteht bei Menschen mit Demenz die Gefahr, für sie unvorteilhafte oder sogar schädliche Entscheidungen zu treffen. Besonders wenn die Entscheidungen schnell getroffen werden müssen und auf vielen und komplexen Informationen basieren, zeigen Menschen mit Demenz klare Nachteile gegenüber Gesunden [42]. In der derzeitigen COVID-19-Krise geraten Menschen mit Demenz besonders schnell und leicht in solch schwierigen Situationen. Auch die komplexe Informationsflut über die Medien und der teils fehlende Austausch mit vertrauten Mitmenschen birgt die Gefahr von potenziell schädlichen Entscheidungen. – Als Beispiel seien nur die häufigen kriminellen Hilfs- oder Kreditangebote, oder gezielte Falschinformationen über COVID-19-Maßnahmen genannt. Es kann davon ausgegangen werden, dass sich viele Menschen mit Demenz allein aufgrund krankheitsbedingter Defizite in der Entscheidungsfindung gegen das Befolgen von Sicherheitsmaßnahmen oder auch das Einhalten von Ausgangsbeschränkungen entschieden haben. Als Gegenmaßnahme wurden verstärkt in den Medien auch Nachrichten „in einfacher Sprache“ gesendet. Auf manchen webbasierten Demenzportalen wurden eigens für Menschen mit Demenz schriftliche und bildliche Erklärungen der erlassenen COVID-19-Maßnahmen veröffentlicht. Wieder wird darauf hingewiesen, dass eben solche Maßnahmen Menschen mit Demenz in besonders fortgeschrittenen Stadien nicht erreichen und ein dringender Bedarf in Krisen besteht, die verfügbaren Informationen für alle Bürger eines Staates verfügbar zu machen.

#### Stellungnahme der ÖAG

Eine aktive und auf Menschen mit Demenz angepasste schriftliche Information, z. B. über Informationsblätter und Broschüren auf staatlicher Ebene, wäre eine sinnvolle und wichtige Initiative.

## Diskussion

Die COVID-19-Pandemie wird mit Sicherheit als bedeutende Krise in die Weltgeschichte und auch die Geschichte Österreichs eingehen. Ob in vielen Jahren der medizinische Aspekt mit tausenden von menschlichen Opfern oder der wirtschaftliche Schaden im Vordergrund bleiben wird, wird die Zukunft zeigen.

Sicher ist jedenfalls, dass Menschen in hohem Lebensalter und damit auch Menschen mit Demenz vornehmlich die Opfer der medizinischen und gesundheitlichen Folgen von COVID-19 sind. Auch steht außer Frage, dass pflegende Angehörige und das Betreuungsumfeld von in der Altenpflege tätigen Personen auch noch nach dieser Krise einem hohen Maß physischer, emotionaler und sozialer Belastung ausgesetzt sind. In welchem Ausmaß die Pandemie die besonders vulnerable Gruppe von geriatrischen und gerontopsychiatrischen Patienten auf den dargestellten Ebenen treffen wird und welche möglichen auch positiven Chancen sich durch die Krise eröffnen, werden die nächsten Monate und Jahre zeigen.

Zum jetzigen Zeitpunkt ist ein wesentlicher Schritt, die COVID-19-Auswirkungen auf möglichst vielen Ebenen zu erheben, zu dokumentieren und kritisch zu beleuchten. Nur so kann auch nach der Krise ein Aufarbeiten der nun akuten und traumatisierenden Situation erfolgen und für präventive Strategien in der Zukunft genutzt werden.

### Stellungnahmen und Empfehlungen der Österreichischen Alzheimer Gesellschaft zur Auswirkung von COVID-19 auf Menschen mit Demenz und deren Betreuungsumfeld

Die Covid-19-Krise führte drastisch vor Augen, wie fragil das österreichische Pflege- und Versorgungssystem von Menschen mit Demenz auf unterschiedlichen Ebenen ist. Die Österreichische Alzheimer Gesellschaft unterstützt deshalb sehr die Aufwertung des Pflegeberufs durch Verbesserungen der Ausbildungsqualität sowie der Entlohnung. Die Autonomie der österreichischen 24-h-Betreuung muss erhöht werden: Dies wird nur über bessere Entlohnung und höhere Wertschätzung möglich sein. Auch eine Ausbildungsoffensive im Bereich der Pflegekräfte muss damit einhergehen. Das derzeitige Pflegesystem zieht seinen Vorteil aus dem unterschiedlichen Lohnniveau innerhalb der Europäischen Union und ist dabei gleichzeitig gewillt bei den qualitativen Mindeststandards Abstriche zu machen. Es ist zu wünschen, dass das Beschwören der „Helden des Alltags“ in vielen Bereichen unserer Gesellschaft, und ganz besonders im Bereich der Pflegeberufe, zu einer Neuorientierung in unserem Land führt. Machen wir uns bewusst, dass die COVID-19-Krise nur ein Katalysator dafür ist, inhärente Systemmängel aufzuzeigen. Die Mängel im Pflegebereich werden uns gerade drastisch vor Augen geführt. Nutzen wir die Chance der sich gerade entwickelnden Werteverschiebungen, um kurz- und mittelfristige Änderungen herbeizuführen. Es ist darauf zu drängen, dass die in den letzten Wochen so viel gepriesenen „systemrelevanten“ Bereiche unserer Gesellschaft – und dazu gehört das Pflegesystem – verstärkt autonomisiert und budgetär adäquat ausgestattet werden müssen. Vor allem jenen Personen in unserem Land, die zu pflegende Angehörige haben, wurde die Verletzlichkeit des derzeitigen Systems drastisch vor Augen geführt.

#### Forderungen und Empfehlungen der ÖAG

- Strukturierte Erfassung von Folgen der COVID-19-Pandemie bei Patienten/-innen mit Demenz und ihrem Betreuungsumfeld über mehrere Jahre.
- Schaffung von Förderungen und finanziellen Ressourcen für die Entwicklung für Krisenpräventionsmaßnahmen für Menschen mit Demenz.
- Ausbau des digitalen Angebots für Menschen mit Demenz und deren Betreuungsumfeld.
- Proaktive Aufklärung und Schulung von professioneller und nicht professioneller Pflege über hygienische Schutzmaßnahmen und zur Verfügung Stellung entsprechender Materialien.
- Autonomie der österreichischen 24-h-Pflege durch bessere Entlohnung und höherer Wertschätzung.

### Zusammenfassung wesentlicher COVID-19-assoziierter Problemfelder, positiver Auswirkungen und forschungsrelevante Fragestellungen

#### Wesentliche aufgetretene Problemfelder mit akutem Handlungsbedarf

- Verfügbarkeit von Schutzmaßnahmen für Pflegepersonen einschließlich pflegender Angehöriger.
- Gewährleistung einer sicheren Betreuung von Menschen mit Demenz mit Pflegekräften vorwiegend aus dem Inland.
- Förderung von digitalen Medien für Menschen mit Demenz.
- Krisenmanagement für Menschen mit Demenz in allen Krankheitsstadien.

#### Positives Resümee

- Die Krise hat gezeigt, dass in Akutsituationen auch neue Hilfsangebote auf allen Ebenen unbürokratisch und schnell geschaffen werden können.

Einige Beispiele dafür sind

- Ehrenamtliche Einkaufsdienste für Senioren
- Aussetzung der chefärztlichen Genehmigungspflicht für Medikamente
- Schnelle, wenn auch befristete Pflegegeldeinstufung
- Telefonische und telemedizinische Versorgung
- Einrichtung von Hotlines

#### Ziele weiterer Forschungsmaßnahmen

- Auswirkungen der Krise auf die psychische und körperliche Gesundheit von Menschen mit Demenz und deren Betreuungsumfeld
- Auswirkungen des COVID-19-Maßnahmengesetzes auf:
  - Progression von kognitiven Defiziten
  - Entwicklung von Suizidraten, Mortalität und Hospitalisierungen

#### Positive Effekte der COVID-19-Krise, die auch nach der Krise bestehen bleiben sollten

- Mehr soziales Engagement in Wohnheimen und Institutionen
- Ehrenamtliche Hilfe für die ältere Bevölkerung im Alltag
- Beibehalt einer verstärkten telemedizinischen und telefonischen medizinischen Versorgung
- Vermeidung von nicht notwendigen Aufnahmen von Menschen mit Demenz im Krankenhaus oder Zuweisungen an Notfallaufnahmen durch Verbesserung des Krisenmanagements
- Telefonhotlines
- Höhere Hygienestandards in Betreuungseinrichtungen und im öffentlichen Bereich
